# Interleukin-40 as a biomarker of mortality risk in patients with severe pneumonia

**DOI:** 10.3389/fimmu.2026.1804357

**Published:** 2026-05-28

**Authors:** Hong Tan, Hanyi Wang, Xuanyi Zhou, Jun Duan, Xiaoliang Yang

**Affiliations:** 1 Department of Blood Transfusion, The First Affiliated Hospital of Chongqing Medical University, Chongqing, China; 2 Key Laboratory of Diagnostic Medicine Designated by the Ministry of Education, Chongqing Medical University, Chongqing, China; 3 Department of Respiratory and Critical Care Medicine, The First Affiliated Hospital of Chongqing Medical University, Chongqing, China

**Keywords:** biomarker, IL-40, mortality, severe pneumonia, SOFA score

## Abstract

Severe pneumonia is an acute lung parenchyma infectious disease with a high incidence and mortality. The role of interleukin-40 (IL-40) in estimating mortality in patients with severe pneumonia remains unknown. This study aims to reveal the clinical application of IL-40 at intensive care unit (ICU) admission as a potentially novel biomarker to predict the mortality risk of severe pneumonia. Patients with severe pneumonia (*n* = 104), patients with non-severe pneumonia (*n* = 50), and healthy controls (*n* = 86) from May 2023 to May 2025 were enrolled. The serum IL-40 levels were measured at admission, and the 28-day survival status of the participants was compared. Furthermore, the area under the receiver operating characteristic curve (AUC) of IL-40 at ICU admission for 28-day survival was used to evaluate the ability of IL-40 in predicting the mortality of severe pneumonia. Kaplan–Meier survival curves were analyzed to evaluate the relationship between IL-40 and the mortality risk of severe pneumonia. IL-40 levels at ICU admission in patients with severe pneumonia were significantly higher than those in patients with non-severe pneumonia and healthy controls. IL-40 levels in non-survivors were significantly higher than those in survivors. The AUC of IL-40 for predicting the mortality risk of severe pneumonia was highest among the indicators. IL-40 and Sequential Organ Failure Assessment (SOFA) scores at ICU admission of patients with severe pneumonia were found to be independent predictors of 28-day mortality. The AUC of IL-40 combined with SOFA score for estimating 28-day mortality in patients with severe pneumonia increased from 0.7626 to 0.7980. Moreover, patients with severe pneumonia with higher IL-40 levels (≥1.244 ng/mL) had poorer survival than those with lower levels (<1.244 ng/mL) (*p* = 0.001). The IL-40 at ICU admission was valuable for predicting the mortality risk of severe pneumonia. These findings can be used as an early biomarker for early clinical decision-making in treating patients with severe pneumonia.

## 1 Introduction

Severe pneumonia, an acute infectious disease involving the lung parenchyma that includes severe community-acquired pneumonia and severe hospital-acquired pneumonia, causes a major global health and economic burden due to its high incidence and mortality (1–3). Severe pneumonia is characterized by rapid progression and frequent complications such as respiratory failure, septic shock, and multiple organ dysfunction, and early treatment is essential to enhance patient survival (4). Therefore, clinical assessment primarily to identify severe pneumonia early relies on various scoring systems and laboratory biomarkers; for instance, the Acute Physiology and Chronic Health Assessment II (APACHE II) scores, the quick Sequential Organ Failure Assessment (qSOFA) scores, the CURB-65 scores, and the Pneumonia Severity Index (PSI) were commonly utilized as tools to predict the likelihood of hospitalization or death in patients with pneumonia (5, 6). However, CURB-65 and PSI had limitations in predicting mortality risk for elderly patients with pneumonia (7). The SOFA score and the APACHE II score were time-consuming and not pneumonia-specific (8, 9). Furthermore, C-reactive protein (CRP), procalcitonin (PCT), and lymphocyte (LYM) remain limitations in non-infectious diseases and severe infections (10, 11).

Indeed, interleukins (ILs) play crucial roles in immune responses and inflammatory processes. Hawerkamp et al. reported that elevated cytokines (IL-1β, IL-2, IL-18, IL-23, IL-33, etc.) and widespread immune dysregulation add further evidence for the role of a pro-inflammatory cytokine signature in severe and critical COVID-19 (12). Available evidence suggests that high levels of IL-6 and IL-28 are associated with the severity of COVID-19, which may be a promising biomarker for detecting disease (13, 14). In addition, IL-6 and IL-10 serve as prevalent sensitive biomarkers for local infections, predicting treatment outcomes and mortality in severe pneumonia, but lacking specificity (15). Therefore, because of the limitations of scoring systems and biomarkers mentioned above, further research on the ILs to find more candidate biomarkers for predicting risk of severe pneumonia mortality has become the focus point (16, 17).

IL-40, a cytokine exclusive to mammals, is encoded by the *C17orf99* gene and plays a crucial role in maintaining IgA secretion, influencing immune responses, and regulating B-cell homeostasis (18). Recently, IL-40 was recognized as a valuable severity indicator of various autoimmune diseases, such as primary Sjogren’s syndrome, rheumatoid arthritis (RA), ankylosing spondylitis (AS), systemic lupus erythematosus (SLE), and type 2 diabetes mellitus (19). However, research on IL-40 in patients with severe pneumonia is a rare. Only a study with a small sample size (only 30 cases) reported elevated IL-40 levels in individuals with COVID-19-related pneumonia, suggesting a potential link with disease severity (20). Nevertheless, whether IL-40 correlates with prognosis in non-COVID severe pneumonia remains unclear. Therefore, the aim of this study is to investigate the relationship between IL-40 and the prognosis of patients with severe pneumonia; meanwhile, we try to assess serum IL-40 concentrations as a potential biomarker for predicting mortality risk in patients with severe pneumonia.

## 2 Materials and methods

### 2.1 Patients and controls

#### 2.1.1 Inclusion and exclusion criteria

All the non-severe pneumonia and severe pneumonia cases enrolled met the diagnostic criteria established by the American Thoracic Society and the Infectious Diseases Society of America (ATS/IDSA). The defining criteria for severe pneumonia included the following: (1) Major criteria: respiratory failure necessitating invasive mechanical ventilation, or septic shock requiring vasopressor therapy despite adequate fluid resuscitation. (2) Minor criteria: respiratory rate ≥30 breaths/min; confusion and/or disorientation; hypothermia (<36 °C); hypotension requiring aggressive fluid resuscitation; leukocyte count <4×10^9^/L; thrombocytopenia (≤100×10^9^/L); blood urea nitrogen ≥20 mg/dL (equivalent to 7.12 mmol/L); PaO_2_/FiO_2_ ratio ≤250 mmHg; and pulmonary multilobar infiltrates (≥2 lobes) (6).

Exclusion criteria were as follows: (1) age < 18 years; (2) presence of malignant tumors; (3) long-term use of immunosuppressants or corticosteroids; (4) HIV-positive status; (5) with complicated infections; (6) with autoimmune diseases; and (7) COVID-19-related pneumonia. All patients were observed for at least 28 days. Based on disease outcomes, patients were categorized into a survival group and a non-survivor group. For the log-rank test, patients were stratified into high IL-40 and low IL-40 groups based on the cutoff value.

#### 2.1.2 Study population

A prospective observational study was performed in the Department of Respiratory and Critical Care Medicine at the First Affiliated Hospital of Chongqing Medical University from May 2023 to May 2025. A total of 186 patients with pneumonia were screened and 32 of them were excluded due to malignant tumors (*n* = 14), long-term using corticosteroids (*n* = 1), complicated infections (*n* = 6), incomplete data (*n* = 4), and autoimmune diseases (*n* = 7). In addition, 86 healthy adults were enrolled as healthy controls. The patients with pneumonia were further stratified into severe (*n* = 104) and non-severe (*n* = 50) pneumonia groups. All the subjects signed informed consent in the study. The study was conducted in accordance with the Declaration of Helsinki, and the protocol was approved by the Ethics Committee of The First Affiliated Hospital of Chongqing Medical University (Approval No. 2023-122).

### 2.2 Clinical parameter collection and biomarker measurement

Clinical parameters including APACHE II score, SOFA score, white blood cell (WBC), CRP, PCT, LYM, and IL-6 were collected on the day of enrollment. At the same time, venous blood samples (4 mL) were obtained from patients. After centrifugation at 3,000 rpm for 5 min at room temperature, the serum was separated and stored at −80 °C. Serum IL-40 concentrations were quantified using an enzyme-linked immunosorbent assay kit (Product code: MBS2905409, mybiosource, USA), and the laboratory operators were blinded to patient outcomes during IL-40 measurement.

### 2.3 Statistical analyses

Statistical analysis was performed by SPSS version 26.0 (IBM Corp, Armonk, NY, USA). Nonnormally distributed variables were presented as medians [interquartile ranges (IQRs)], and the differences between groups were tested by the Kruskal–Wallis test, the nonparametric Mann–Whitney *U* test, or the Wilcoxon matched-pairs signed rank test, as appropriate. To evaluate the correlation between IL-40 and APACHE II score, SOFA score, CRP, PCT, and IL-6, Spearman’s correlation coefficients were calculated to conduct the correlation analysis, and all data were checked for normality using the Shapiro–Wilk test. To estimate the value of IL-40 for predicting 28-day mortality in patients with severe pneumonia, the ROC curve was constructed by IL-40 levels at admission. The DeLong test was used for comparison of AUC. The cutoff values were calculated corresponding to the maximum value of the Youden index of ROC. The Kaplan–Meier survival rates were analyzed to evaluate the relationship between IL-40 expression and the mortality risk of severe pneumonia. Logistic regression analysis, multicollinearity analysis, and multiple binary logistic regression were used to determine the independent predictors of mortality. All analyses were two-sided, and statistical significance was set at *p* < 0.05.

## 3 Results

### 3.1 Patient clinical features

A cohort comprising 104 patients with severe pneumonia, 50 patients with non-severe pneumonia, and 86 healthy controls was enrolled, as depicted in **Supplementary Figure 1**. The basic demographics and clinical characteristics of these subjects are summarized in **Supplementary Table 1**. There were no differences in sex, age, WBC, and CRP between patients with severe pneumonia and patients with non-severe pneumonia. The levels of PCT (*p* < 0.0001), APACHE II score (*p* < 0.0001), SOFA score (*p* < 0.0001), mortality (*p* < 0.0001), chronic disease combined organ dysfunction (*p* < 0.0001), and miscellaneous infection (*p* < 0.0001) in the severe pneumonia groups were higher within 24 h after intensive care unit (ICU) admission than those in the non-severe pneumonia groups. However, comorbidities (*p* = 0.002) in the severe pneumonia groups were lower than those in the non-severe pneumonia groups.

### 3.2 Serum IL-40 levels in patients with severe pneumonia

**Figure 1:**
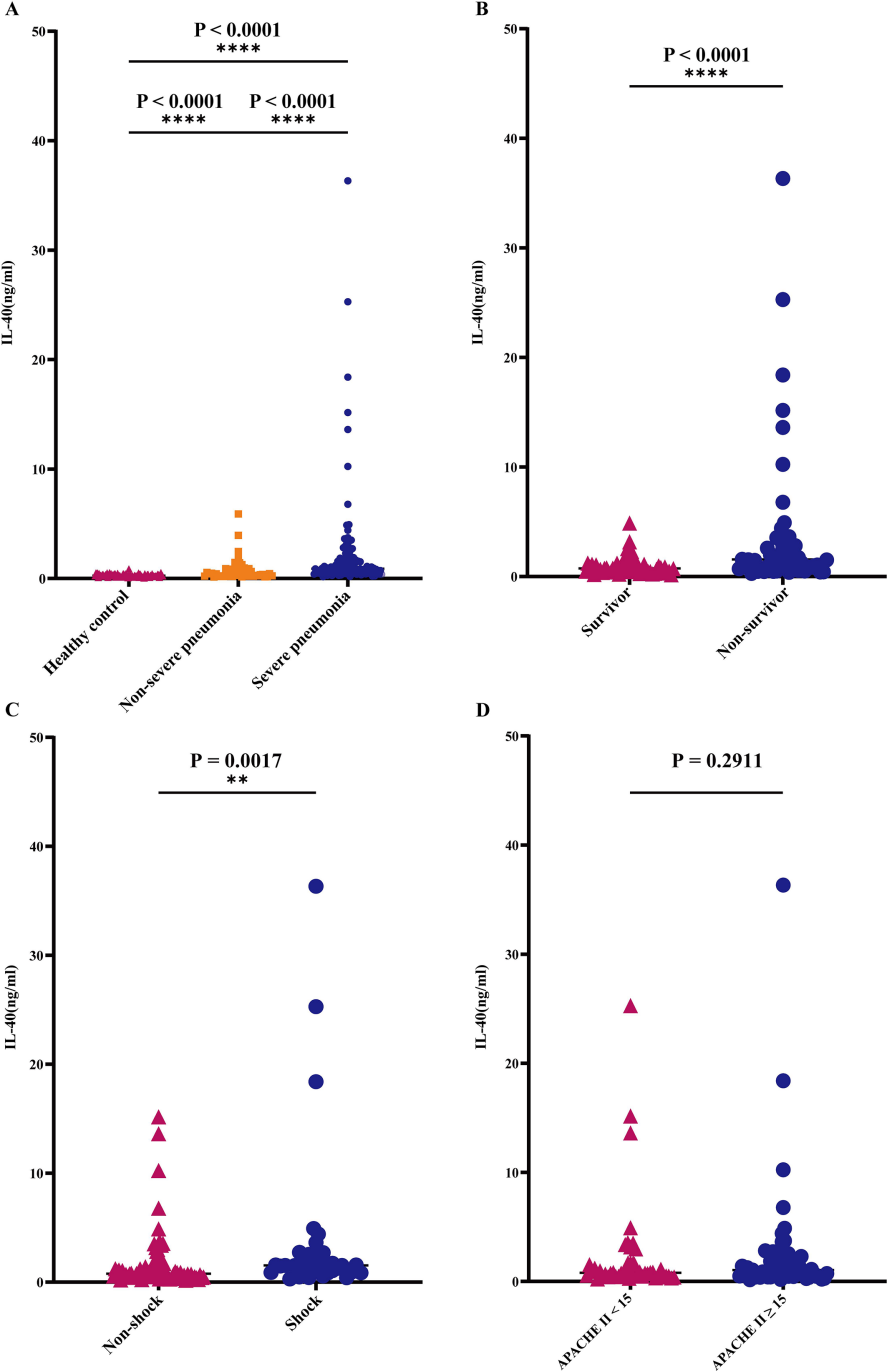
**(A)** Serum IL-40 was elevated in patients with severe pneumonia. Serum IL-40 levels were detected by ELISA from 104 patients with severe pneumonia, 50 patients with non-severe pneumonia, and 86 healthy controls. **(B)** Serum IL-40 levels of non-survivors and survivors in patients with severe pneumonia. **(C)** Serum IL-40 levels of patients with severe pneumonia with shock and non-shock. **(D)** Serum IL-40 levels of patients with severe pneumonia were grouped by an APACHE II score of 15. Statistical analysis was performed with the nonparametric Kruskal–Wallis test **(A)** and Mann–Whitney *U* test **(B–D)**; *p* ≤ 0.05 was considered statistically significant.

The serum IL-40 levels of patients with severe pneumonia were significantly higher than those of patients with non-severe pneumonia (*p* < 0.0001) and healthy control groups (*p* < 0.0001). In contrast, the IL-40 levels of patients with non-severe pneumonia were also significantly higher than those of healthy control groups (*p* < 0.0001), as shown in **Figure 1A**. The expression of IL-40 in severe pneumonia non-survivors was higher than those in survivors at admission (*p* < 0.0001), as shown in **Figure 1B**. The expression of IL-40 in patients with severe pneumonia with shock was higher than that of patients without shock at admission (*p* = 0.0017), as shown in **Figure 1C**. No statistical difference was observed in patients with severe pneumonia based on an APACHE II score cutoff of 15 (*p* = 0.2911), as shown in **Figure 1D**.

### 3.3 Correlation of serum IL-40 and severe pneumonia assessment indicators

**Figure 2:**
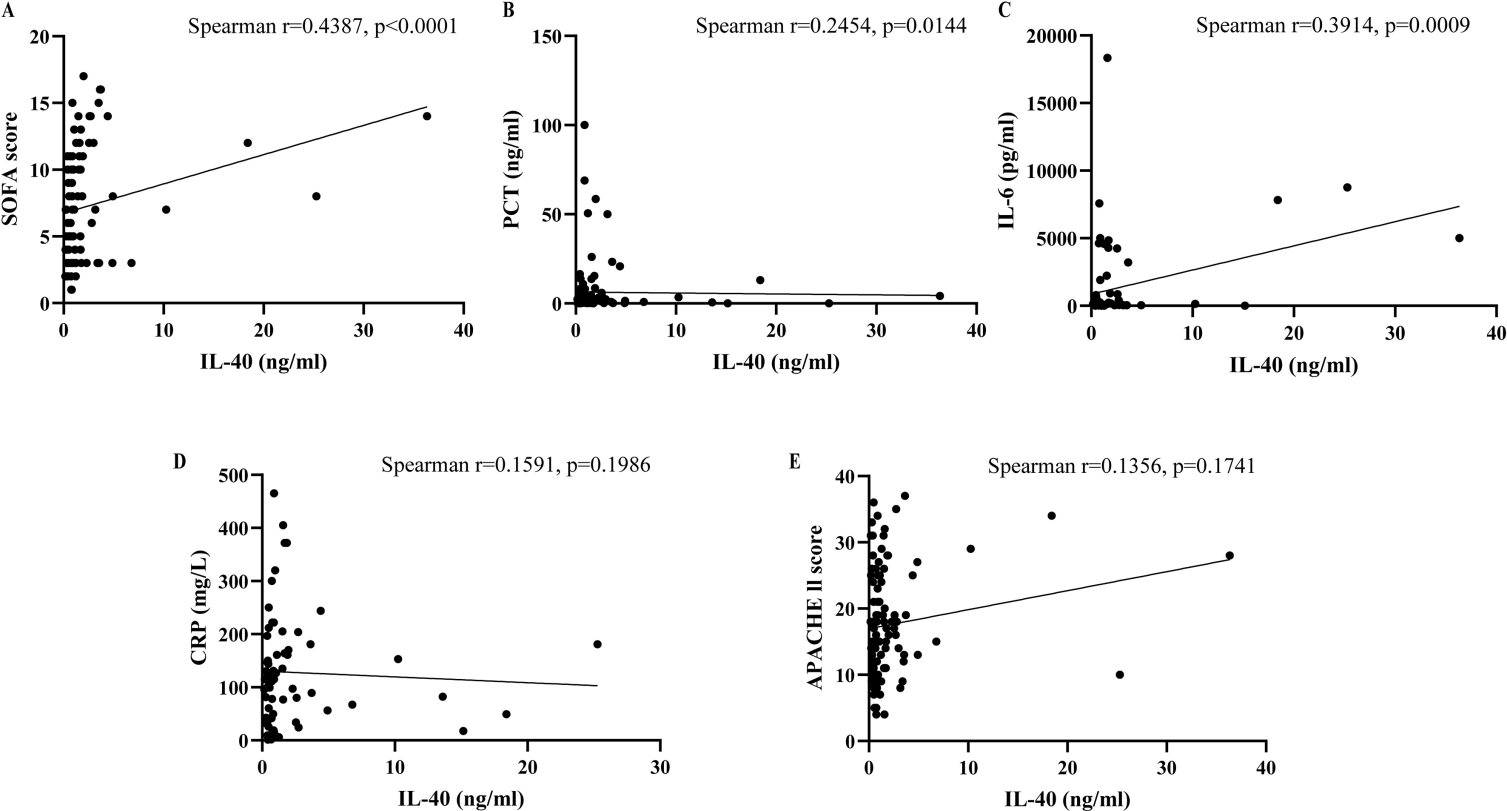
Serum IL-40 was correlated with indicators in patients with severe pneumonia. **(A)** Serum IL-40 levels were positively correlated with SOFA scores (*r* = 0.4387, *p* < 0.0001). **(B)** Serum IL-40 levels were positively correlated with serum PCT levels (*r* = 0.2454, *p* = 0.0144). **(C)** Serum IL-40 levels were positively correlated with serum IL-6 levels (*r* = 0.3914, *p* = 0.0009). **(D)** Serum IL-40 levels were not significantly correlated with serum CRP levels (*r* = 0.1591, *p* = 0.1986). **(E)** Serum IL-40 levels were not significantly correlated with APACHE II scores (*r* = 0.1356, *p* = 0.1741). Statistical analysis was performed with Spearman’s correlation coefficient test.

The correlations between IL-40 and severe pneumonia assessment indicators (APACHE II score, SOFA score, IL-6, PCT, and CRP) are shown in **Figures 2A–E**. The results displayed that serum IL-40 levels were not significantly correlated with APACHE II scores and CRP. However, serum IL-40 levels were positively correlated with SOFA scores, IL-6, and PCT, with correlation coefficients of 0.4387 (*p* < 0.0001), 0.3914 (*p* = 0.0009), and 0.2454 (*p* = 0.0144), respectively.

### 3.4 ROC curves evaluating the ability of IL-40 to predict mortality of severe pneumonia

**Figure 3:**
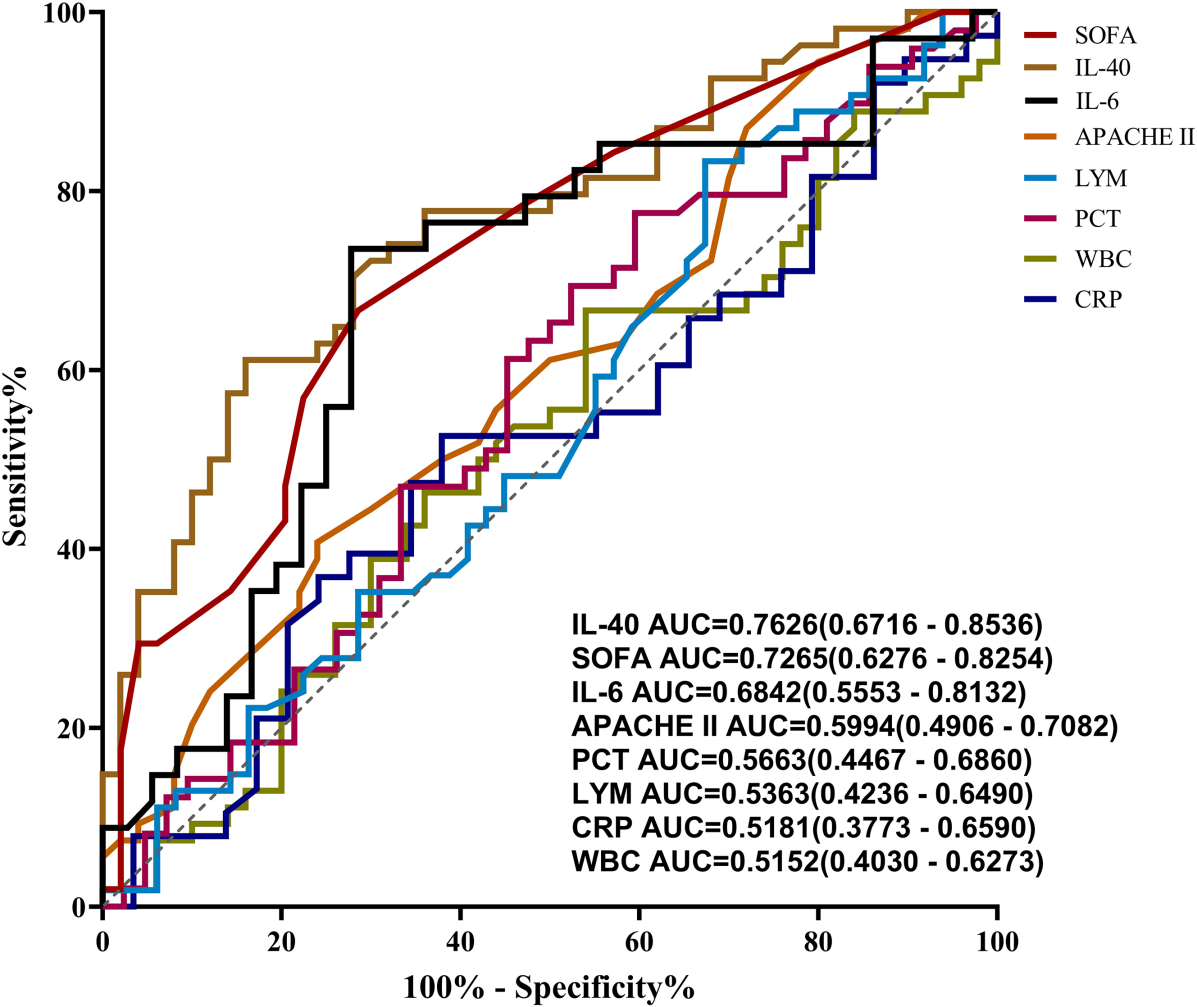
Efficacy of each indicators in predicting mortality by ROC curve analysis.

To evaluate the predictive value of IL-40 for mortality, the AUCs of IL-40, SOFA score, IL-6, APACHE II score, PCT, LYM, CRP, and WBC were calculated by ROC curves. The AUC of IL-40 (0.7626, 95% CI: 0.6716–0.8536) for predicting severe pneumonia mortality was higher than SOFA (0.7265, 95% CI: 0.6276–0.8254), IL-6 (0.6842, 95% CI: 0.5553–0.8132), APACHE II (0.5994, 95% CI: 0.4906–0.7082), PCT (0.5663, 95% CI: 0.4467–0.6860), LYM (0.5363, 95% CI: 0.4236–0.6490), CRP (0.5181, 95% CI: 0.3773–0.6590), and WBC (0.5152, 95% CI: 0.4030–0.6273). Compared with ROC of IL-40, APACHE II (*p* = 0.020), PCT (*p* = 0.003), LYM (*p* < 0.001), CRP (*p* < 0.001), and WBC (*p* < 0.001) showed statistically significant differences except for SOFA (*p* = 0.697) and IL-6 (*p* = 0.246) analyzed by the DeLong test. Moreover, the cutoff value, SE, SP, PPV, and NPV of IL-40, SOFA score, IL-6, APACHE II score, PCT, LYM, CRP, and WBC for predicting severe mortality were as follows: IL-40 (1.244, 61.11%, 84.00%, 80.49%, and 66.67%), SOFA (6.5, 66.67%, 71.43%, 70.83%, and 72.58%), IL-6 (131, 73.53%, 72.22%, 71.43%, and 74.29%), APACHE II (19.5, 40.74%, 76.00%, 64.71%, and 54.29%), PCT (0.315, 77.55%, 40.48%, 60.32%, and 60.71%), LYM (1.115, 83.33%, 32.65%, 36.00%, and 42.31%), CRP (124.5, 52.63%, 62.07%, 64.52%, and 50.00%), and WBC (9.205, 66.67%, 46.00%, 57.14%, and 56.10%), respectively, as shown in **Figure 3** and **Table 1**.

**Table 1:** The AUC, optimal cutoff values, and validity indexes of optimal research parameters for predicting severe pneumonia mortality.

Parameter	Cutoff value	AUC (95% CI)	SE (%)	SP (%)	PPV (%)	NPV (%)	Youden index (%)	*p*-value (compared with IL-40)
IL-40	1.244	0.7626 (0.6716,0.8536)	61.11	84.00	80.49	66.67	45.11	–
SOFA	6.5	0.7265 (0.6276,0.8254)	66.67	71.43	70.83	72.58	38.10	0.697
IL-6	131	0.6842 (0.5553,0.8132)	73.53	72.22	71.43	74.29	45.75	0.246
APACHE II	19.5	0.5994 (0.4906,0.7082)	40.74	76.00	64.71	54.29	16.74	0.020*
PCT	0.315	0.5663 (0.4467,0.6860)	77.55	40.48	60.32	60.71	18.03	0.003**
LYM	1.115	0.5363 (0.4236,0.6490)	83.33	32.65	36.00	42.31	15.98	0.0001***
CRP	124.5	0.5181 (0.3773,0.6590)	52.63	62.07	64.52	50.00	14.70	0.0006***
WBC	9.205	0.5152 (0.4030,0.6273)	66.67	46.00	57.14	56.10	12.67	0.0008***

AUC, area under the curve; SOFA, Sequential Organ Failure Assessment Score; LYM, lymphocyte; APACHE II, Acute Physiology and Chronic Health Evaluation II; WBC, white blood cell; PCT, procalcitonin; SE, sensitivity; SP, specificity; PPV, positive predictive value; NPV, negative predictive value. *p < 0.05, **p < 0.01, ***p < 0.001.

### 3.5 Independent predictors of severe pneumonia mortality

The results of the univariate analysis displayed that age (β = 0.033, OR = 1.034, 95% CI: 1.001–1.067, *p* = 0.042), IL-40 levels (β = 0.793, OR = 2.210, 95% CI: 1.343–3.636, *p* = 0.002), and the SOFA score (β = 0.215, OR = 1.240, 95% CI: 1.105–1.390, *p* < 0.001) were significantly associated with patient mortality according to binary logistic regression, whereas sex, comorbidities, APACHE II score, WBC, LYM, CRP, PCT, and IL-6 had no significant effect on mortality, as shown in **Table 2**. Before multivariate regression analysis, multi-collinearity diagnosis was carried out. The results showed that no multicollinearity was observed in all indicators, as shown in **Supplementary Table 2**. Furthermore, the results of multivariate binary logistic regression analysis showed that only IL-40 levels (*B* = 0.604, OR = 1.830, 95% CI: 1.130–2.963, *p* = 0.014) and SOFA scores (*B* = 0.231, OR = 1.260, 95% CI: 1.086–1.460, *p* = 0.002) on the day of admission were independent predictors of 28-day mortality for patients with severe pneumonia, as shown in **Table 3**.

**Table 2:** Univariate logistic regression analysis to evaluate the effect of each indicator on the mortality of patients with severe pneumonia.

Parameter	β	SE	Wald	Degrees of freedom	*p*-value	Odds ratio	95% confidence interval
Age	0.033	0.016	4.152	1	0.042*	1.034	1.001 -1.067
Sex	0.395	0.440	0.807	1	0.369	1.484	0.627–3.513
CD	0.095	0.544	0.031	1	0.861	1.100	0.379–3.196
OD	0.788	0.664	1.410	1	0.235	2.200	0.599–8.084
CD+OD	0.021	0.582	0.001	1	0.971	1.021	0.326–3.199
IL-40	0.793	0.254	9.738	1	0.002**	2.210	1.343–3.636
APACHE II	0.044	0.025	3.238	1	0.072	1.045	0.996–1.097
SOFA	0.215	0.059	13.46	1	<0.001***	1.240	1.105–1.390
WBC	−0.005	0.027	0.31	1	0.860	0.995	0.945–1.049
LYM	−0.395	0.320	1.522	1	0.217	0.674	0.360–1.261
CRP	0.000	0.002	0.006	1	0.941	1.000	0.995–1.005
PCT	−0.015	0.015	1.044	1	0.307	0.985	0.957–1.014
IL-6	0.000	0.000	1.787	1	0.181	1.000	1.000–1.000

IL-40, interleukin-40; APACHE II, Acute Physiology and Chronic Health Evaluation II; SOFA, Sequential Organ Failure Assessment; WBC, white blood cell; LYM, lymphocyte; CRP, C-reactive protein; PCT, procalcitonin; IL-6, interleukin-6; CD, chronic disease, including high blood pressure, coronary heart disease, diabetes; OD, organ dysfunction including heart failure, liver and kidney dysfunction. *p < 0.05, **p < 0.01, ***p < 0.001.

**Table 3:** Multivariable binary logistic regression to analyze the independent predictors of 28-day mortality of patients with severe pneumonia.

Parameter	β	SE	Wald	Degrees of freedom	*p*-value	Odds ratio	95% confidence interval
Age	0.036	0.020	3.371	1	0.066	1.037	0.998–1.077
Sex	0.315	0.530	0.352	1	0.553	1.370	0.484–3.875
CD	−0.613	0.692	0.783	1	0.376	0.542	0.140–2.105
OD	−0.338	0.882	0.147	1	0.701	0.713	0.127–4.015
CD+OD	−1.466	0.798	3.372	1	0.066	0.231	0.048–1.104
IL-40	0.604	0.246	6.043	1	0.014*	1.830	1.130–2.963
SOFA	0.231	0.075	9.351	1	0.002**	1.260	1.086–1.460

IL-40, interleukin-40; APACHE II, Acute Physiology and Chronic Health Evaluation II; SOFA, Sequential Organ Failure Assessment; WBC, white blood cell; LYM, lymphocyte; CRP, C-reactive protein; PCT, procalcitonin; IL-6, interleukin-6; CD, chronic disease, including high blood pressure, coronary heart disease, diabetes; OD, organ dysfunction including heart failure, liver and kidney dysfunction. *p < 0.05, **p < 0.01.

### 3.6 Combined efficacy of IL-40 with SOFA score

In order to further investigate whether it could improve the prediction ability of severe pneumonia mortality, IL-40 combined with SOFA score was evaluated. The AUC of IL-40 combined with SOFA score for estimating severe pneumonia mortality increased significantly from 0.7626 (95% CI: 0.6716–0.8536, *p* < 0.0001) to 0.7980 (95% CI: 0.712–0.884), as shown in **Supplementary Figure 2** and **Table 4**.

**Table 4:** The combination of IL-40 with SOFA for predicting severe pneumonia mortality.

Parameter	AUC (95% CI)	SE (%)	SP (%)	PPV (%)	NPV (%)	Youden index (%)
IL-40+SOFA	0.798 (0.712,0.884)	80.4	67.3	71.89	76.74	47.7

IL-40, interleukin-40; AUC, area under the curve; SOFA, Sequential Organ Failure Assessment Score; SE, sensitivity; SP, specificity; PPV, positive predictive value; NPV, negative predictive value.

### 3.7 Kaplan–Meier curves for severe pneumonia survival according to IL-40 levels

**Figure 4:**
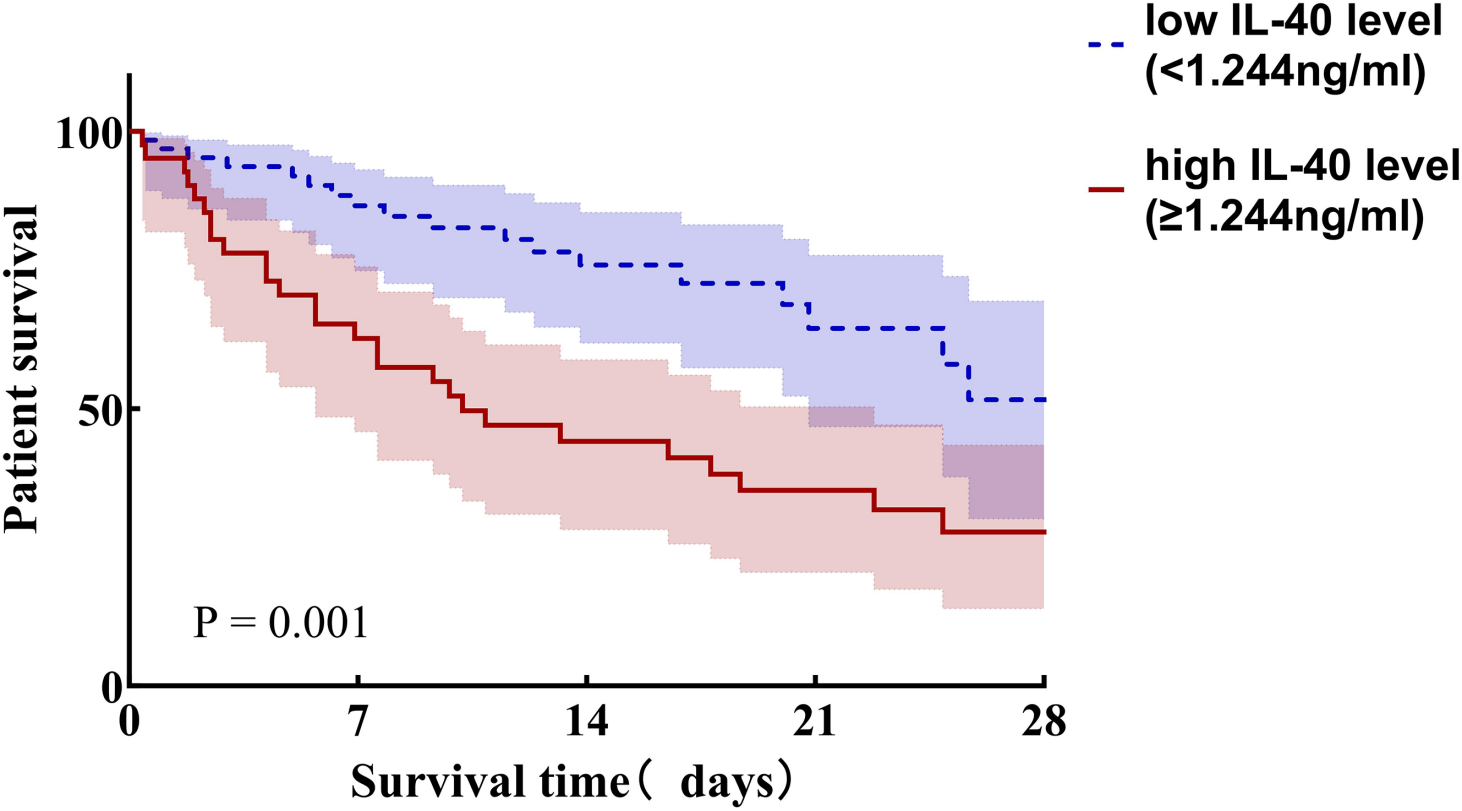
Kaplan–Meier survival curves for IL-40 levels in patients with severe pneumonia. The cutoff value of IL-40 for predicting 28-day mortality in patients with severe pneumonia was 1.244 ng/mL.

To estimate the survival situation of IL-40 for the risk of severe pneumonia 28-day mortality, Kaplan–Meier survival curve was performed. Patients were divided into high- and low-level IL-40 groups using the optimal cutoff value (1.244) obtained from the ROC curve analysis for predicting mortality, as shown in **Table 2**. According to Kaplan–Meier survival curve analysis, the risk of mortality in patients with severe pneumonia with higher IL-40 levels (≥1.244) was 2.795 times that of patients with severe pneumonia with lower IL-40 levels (<1.244). The results revealed that patients with severe pneumonia with higher IL-40 levels had poorer survival than those with lower IL-40 levels (*p* = 0.001), as shown in **Figure 4**.

## 4 Discussion

This study demonstrated serum IL-40 as a novel and independent prognostic biomarker in severe pneumonia. Several key findings were displayed: patients with severe pneumonia exhibited significantly higher IL-40 levels than those with non-severe pneumonia or healthy controls; non-survivors had significantly higher levels than survivors among patients with severe pneumonia; IL-40 levels were correlated positively with SOFA scores, IL-6, and PCT, underscoring its association with disease severity and systemic inflammation; IL-40 demonstrated superior predictive accuracy for mortality (AUC = 0.7626) over traditional biomarkers; patients with higher IL-40 levels (≥1.244) at admission were at a significantly increased risk of 28-day mortality. Moreover, multivariate analysis confirmed IL-40 as an independent predictor of mortality, and its integration with the SOFA score significantly enhanced prognostic performance (AUC = 0.7980). To our knowledge, it was the first study to validate the clinical utility of IL-40 in the prognostic prediction of severe pneumonia, offering a complementary tool that reflects the intertwined inflammatory and dysregulated immune responses characteristic of the disease.

IL-40, the most recently identified B cell-associated member of the interleukin family, has emerged as an important mediator of immune regulation and inflammatory responses. Multiple studies have demonstrated elevated serum IL-40 concentrations in patients with autoimmune diseases such as RA, primary Sjögren’s syndrome (pSS), AS, and SLE (21–25). Moreover, IL-40 levels correlate closely with disease activity in both pSS and SLE (22, 23). However, research investigating IL-40 in acute infectious diseases and inflammation-related conditions remains relatively limited. Recently, Bagriacik et al. reported increased serum IL-40 concentrations in patients with COVID-19 pneumonia and posited correlations between IL-40, IgA, and biomarkers associated with a regulated cell death process known as NETosis (20). Although this study provided initial evidence for IL-40’s role in infectious respiratory diseases, the small sample size (only 30 cases) and the unique pathophysiology of COVID-19 limited the generalizability of these findings to other forms of severe pneumonia. Moreover, an investigation in patients with sepsis has shown that elevated IL-40 levels across various infection sources, including respiratory tract infections, could serve as a potential marker of systemic inflammation in severe infections (26). Importantly, for the first time, our study with a larger sample size demonstrated that higher IL-40 levels in patients with severe pneumonia were associated with increased disease severity and worse clinical outcomes, which was consistent with the observations mentioned above in acute infectious diseases.

Severe pneumonia represents a critical clinical challenge characterized by rapid disease progression, high morbidity and mortality rates, and substantial healthcare resource utilization (27). Furthermore, the heterogeneous nature of severe pneumonia, involving diverse causative pathogens, variable host immune responses, and differing baseline comorbidities, makes accurate prognostic assessment crucial for optimal clinical decision-making (28). Therefore, early identification of patients at high risk for poor outcomes and the development and validation of reliable prognostic markers are essential for optimizing treatment strategies and resource allocation. Current prognostic assessment in severe pneumonia relies on clinical scoring systems and laboratory biomarkers, each with inherent limitations. PSI, the scoring system recommended by the ATS/IDSA for severe pneumonia prognosis, is too complex for practical application in the emergency department (6, 29). By contrast, serum IL-40 can be detected by ELISA or FCM, which is a simple and reproducible measurement. Furthermore, CRP and PCT, the most common indicators of systemic inflammation and bacterial infection, showed no association between a single measurement at admission and mortality in patients with severe pneumonia (30). In our study, miscellaneous infection rates in the severe pneumonia group, which often happened in patients at the ICU, were higher than those in the non-severe pneumonia group. The expression of IL-40 in both severe pneumonia non-survivors and patients with shock was higher than that of controls at admission, which revealed that the expression of IL-40 was positively correlated with the severity of severe pneumonia. Also, the IL-40 levels were also positively correlated with inflammatory cytokines (IL-6) and infection-related biomarkers (PCT), underscoring its relevance to disease progression. The superiority of IL-40 over conventional biomarkers may be attributed to its specific involvement in B cell-mediated immunity and NETosis, both of which are critical for pathogen clearance in pneumonia (31, 32), which could be the basis of IL-40 as a convenient and rapid alternative to prognostic scoring systems.

The condition of severe pneumonia frequently leads to life-threatening complications, with mortality rates ranging from 27% to 50%, despite advances in critical care management (4). Therefore, suitable predictive and prognostic biomarkers are essential for optimizing treatment strategies and improving outcomes. Our study demonstrated that IL-40 achieved superior predictive prognosis ability with an AUC of 0.7626 higher than that in sepsis (AUC = 0.7266) (26), significantly outperforming traditional biomarkers including CRP, PCT, and lymphocyte count. Interestingly, the AUC of IL-40 was also higher than that in IL-6 and SOFA score, but no differences were shown from the two indicators after the DeLong test. Also, the results provided good specificity (84.00%), maintaining reasonable sensitivity (61.11%) and better positive predictive value (80.49%). This enhanced predictive accuracy translates to more reliable identification of high-risk patients requiring intensive monitoring and aggressive intervention. Meanwhile, Kaplan–Meier survival analysis confirmed that patients with IL-40 levels ≥1.244 ng/mL had a 2.795-fold increased risk of 28-day mortality compared to those with lower levels. Together, the results reveal a marked increase in IL-40 in patients with severe pneumonia and its value as a novel potential prognostic biomarker for outcomes.

Interestingly, we found that combining IL-40 with the SOFA score further improved predictive performance, with the AUC increasing from 0.7626 to 0.7980 in our study. Severe pneumonia is characterized by a dynamic and intricate process in which poor prognosis is linked to systemic inflammatory response syndrome, which is characterized by excessive cytokine release, complement activation, neutrophil infiltration, and dysregulated immune responses leading to inflammatory storm and organ dysfunction (33, 34). IL-40 levels correlated positively with IL-6 and PCT, suggesting that it participates in or marks the intensity of systemic inflammation. In contrast, the SOFA score was frequently employed to assess the extent and severity of organ dysfunction across multiple systems in infectious diseases (35). In addition, logistic regression confirmed that both IL-40 and SOFA were independent predictors of mortality. Thus, the combination of IL-40 and SOFA score provides a more comprehensive assessment encompassing the upstream inflammatory response and the downstream physiological impact. Moreover, the combination addresses the problem for temporal dynamics of severe pneumonia progression. IL-40 may increase early in the inflammatory response, potentially before overt organ dysfunction develops. The SOFA score may lag behind the inflammatory cascade. By capturing both early warning signals and established severity, the combined model improves risk stratification across the disease spectrum. This is particularly valuable in severe pneumonia, where the window for effective intervention is narrow and early identification of high-risk patients is crucial. Based on the above advantages, serum IL-40 may be a valuable predictor of severe pneumonia mortality and is expected to be used in patients.

Despite the findings, our study includes some limitations. First, this was a single-center study, which may limit the generalizability of our findings to other healthcare settings with different patient populations and practice patterns. Multicenter prospective studies involving diverse healthcare settings are needed to validate our findings. Second, our inclusion criteria were restricted to adult cases. Whether the prognostic utility of IL-40 extends to pediatric severe pneumonia remains to be elucidated and is a priority for our subsequent research. Third, computed tomography (CT) or chest imaging data could display the disease severity visually, which was not considered in this study. We will explore the relationship with disease severity and IL-40 levels including imaging data in our further study. Fourth, the cutoff value of IL-40 was derived from the same cohort using ROC curve analysis and then applied for survival analysis, which may introduce optimism bias and potentially overestimate its prognostic performance. Therefore, external validation or internal cross-validation is warranted to confirm the true prognostic value of IL-40 in our further study. Finally, the mechanistic basis by which IL-40 contributes to severe pneumonia pathophysiology remains unexplored. Future studies incorporating mechanistic investigations would provide a deeper understanding of the role of IL-40 in severe pneumonia.

## 5 Conclusion

Our study found that serum IL-40 levels at admission were valuable for predicting the 28-day mortality risk of patients with severe pneumonia, which suggests that IL-40 may be a novel biomarker that can be used to identify a group of patients with severe pneumonia who have a higher risk of mortality. These findings can be used for early clinical decision-making in treating patients with severe pneumonia.

## Data Availability

The original contributions presented in the study are included in the article/**Supplementary Material**. Further inquiries can be directed to the corresponding authors.
