# Supplementary material for: Interleukin-40 as a biomarker of mortality risk in patients with severe pneumonia
Source: Front Immunol. 2026 May 28;17:1804357. doi: 10.3389/fimmu.2026.1804357 (PMC13252780; doi:10.3389/fimmu.2026.1804357)
Supplement: Supplementary file 1 [file DataSheet1.doc]

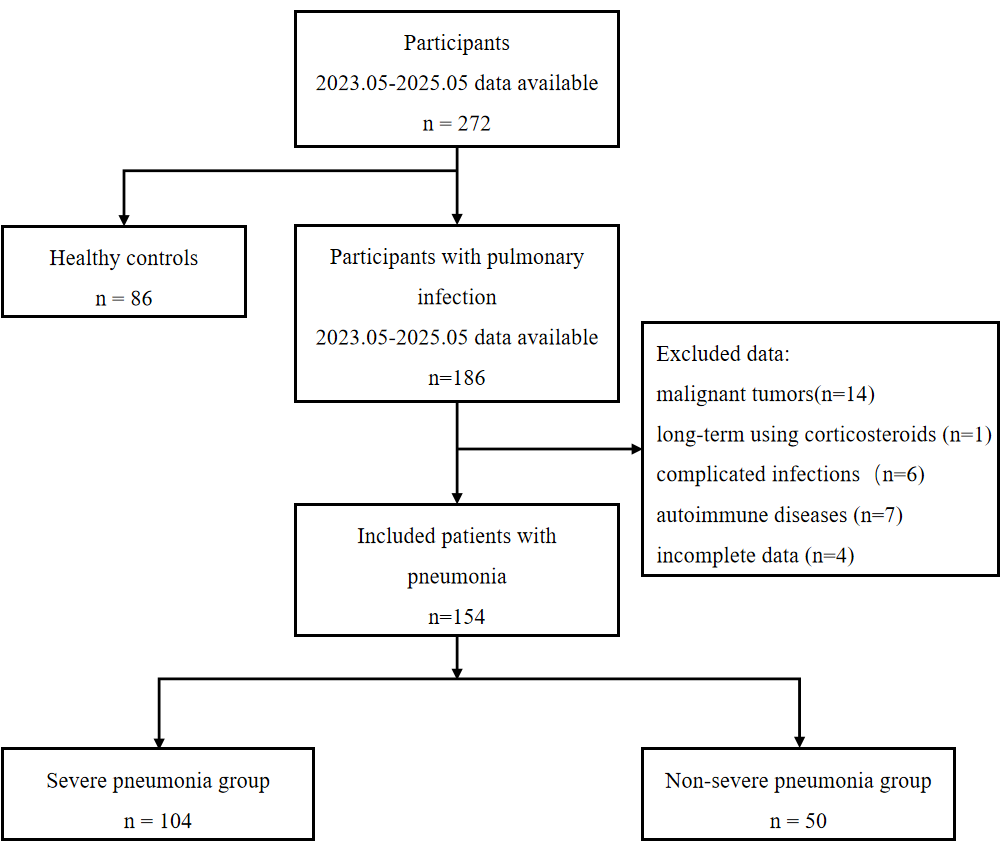


**Figure S1**. Flow chart of study selection.


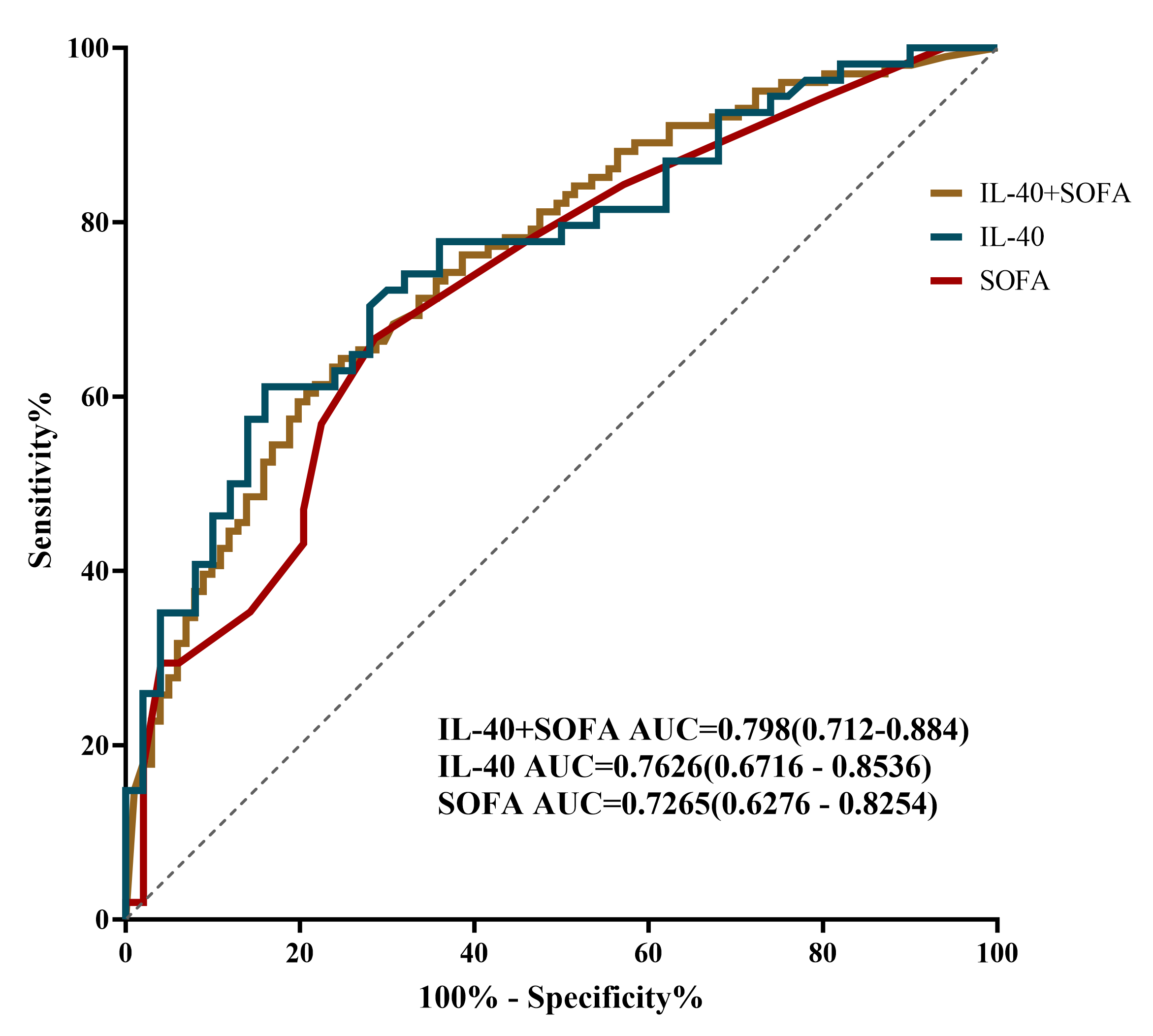


**Figure S2. Efficacy of IL-40, SOFA, and IL-40 combined with SOFA for predicting severe pneumonia** mortality by ROC curve analysis**.**

**Table S1.** Characteristics of severe pneumonia, non-severe pneumonia and healthy controls

| **Characteristics** | **Severe pneumonia**  **(n=104)** | **Non-severe pneumonia**  **(n=50)** | **Healthy controls**  **(n=86)** | ***P* value** | |
| --- | --- | --- | --- | --- | --- |
| Male sex | 75 (72.1%) | 31 (62.0%) | 56 (65.1%) | 0.295 | |
| Age, years | 71 (61.0,78.8) | 71.5 (57.3, 82.8) | 61.5 (53,70) | 0.486 | |
| WBC, 10^9^/L | 10.84 (7.348,14.33) | 8.46 (7.150,10.62) | NA | 0.122 | |
| CRP, mg/L | 113.0 (47.55,165.5) | 45 (27.15,154.9) | NA | 0.106 | |
| PCT, mg/L | 0.947 (0.201,4.083) | 0.12 (0.04,0.31) | NA | ＜0.0001**** | |
| APACHE II score | 15.5 (11.0,25.0) | 5.793 (4.326,7.261) | NA | ＜0.0001**** | |
| SOFA score | 6 (3.0,10.75) | 0.931 (0.4772-1.385) | NA | ＜0.0001**** | |
| ICU stay, days | 8.2 (4.05,16.45) | NA | NA | - | |
| Mortality | 54（51.9%） | 3（6.0%） | NA | ＜0.0001**** | |
| Comorbidities | | | | | |
| CD | 38 (36.5%) | 24 (48.0%) | NA | 0.174 | |
| OD | 18 (17.3%) | 3 (6.0%) | NA | 0.056 | |
| CD+OD | 27 (26.0%) | 1 (2.0%) | NA | ＜0.0001**** | |
| None | 21 (21.2%) | 22 (44.0%) | NA | 0.002** | |
| Isolates, no. of patients | | | | | |
| Gram positive | 9 (8.7%) | 3 (6.0%) | NA | 0.565 | |
| Gram negative | 15 (14.4%) | 3 (6.0%) | NA | 0.128 | |
| Fungus | 12 (11.5%) | 4 (8.0%) | NA | 0.500 | |
| Virus | 4 (3.8%) | 2 (4.0%) | NA | 0.963 | |
| Atypical pathogen | 3 (2.9%) | 5 (10.0%) | NA | 0.062 | |
| Tuberculosis | 3 (2.9%) | 2 (4.0%) | NA | 0.715 | |
| Miscellaneous | 51 (49%) | 2 (4.0%) | NA | ＜0.0001**** | |
| Unknown | 8 (7.7%) | 29 (58.0%) | NA | ＜0.0001**** | |
| NOTE. Data are expressed as medians (interquartile ranges) unless otherwise indicated. APACHE II: acute physiology and chronic health evaluation II; SOFA: sequential organ failure assessment; ICU: intensive care unit; WBC: white blood cell; CRP: C-reactive protein; PCT: procalcitonin; CD: chronic disease including high blood pressure, coronary heart disease, diabetes; OD: organ dysfunction including heart failure, liver and kidney dysfunction. **P*<0.05, ***P*<0.01, ****P*<0.001, *****P*<0.0001. | | | | |  |

**Table S2. Multicollinearity analysis to evaluate the effect of each indicator on the mortality of severe pneumonia patients.**

| **Parameter** | **Tolerance** | **VIF** |
| --- | --- | --- |
| Age | 0.805 | 1.243 |
| IL-40 | 0.677 | 1.478 |
| APACHE II | 0.659 | 1.518 |
| SOFA | 0.600 | 1.667 |
| WBC | 0.616 | 1.624 |
| LYM | 0.711 | 1.406 |
| CRP | 0.579 | 1.726 |
| PCT | 0.453 | 2.207 |
| IL-6 | 0.559 | 1.788 |

NOTE: IL-40: interleukin-40; APACHE II: acute physiology and chronic health evaluation II; SOFA: sequential organ failure assessment; WBC: white blood cell; LYM: lymphocyte; CRP: C-reactive protein; PCT: procalcitonin; IL-6: interleukin-6; VIF:variance inflation factor; VIF < 10 indicates no significant multicollinearity.
